# Transcription factors in microalgae: genome-wide prediction and comparative analysis

**DOI:** 10.1186/s12864-016-2610-9

**Published:** 2016-04-11

**Authors:** Stanislas Thiriet-Rupert, Grégory Carrier, Benoît Chénais, Camille Trottier, Gaël Bougaran, Jean-Paul Cadoret, Benoît Schoefs, Bruno Saint-Jean

**Affiliations:** IFREMER, Physiology and Biotechnology of Algae Laboratory, rue de l’Ile d’Yeu, 44311 Nantes, France; MicroMar, Mer Molécules Santé, IUML - FR 3473 CNRS, University of Le Mans, Le Mans, France

**Keywords:** Algae, Endosymbiotic gene transfer, Haptophytes, Prediction pipeline, Stramenopiles, *Tisochrysis lutea*, Transcription factors

## Abstract

**Background:**

Studying transcription factors, which are some of the key players in gene expression, is of outstanding interest for the investigation of the evolutionary history of organisms through lineage-specific features. In this study we performed the first genome-wide TF identification and comparison between haptophytes and other algal lineages.

**Results:**

For TF identification and classification, we created a comprehensive pipeline using a combination of BLAST, HMMER and InterProScan software. The accuracy evaluation of the pipeline shows its applicability for every alga, plant and cyanobacterium, with very good PPV and sensitivity. This pipeline allowed us to identify and classified the transcription factor complement of the three haptophytes *Tisochrysis lutea*, *Emiliania huxleyi* and *Pavlova* sp.; the two stramenopiles *Phaeodactylum tricornutum* and *Nannochloropsis gaditana*; the chlorophyte *Chlamydomonas reinhardtii* and the rhodophyte *Porphyridium purpureum*. By using *T. lutea* and *Porphyridium purpureum*, this work extends the variety of species included in such comparative studies, allowing the detection and detailed study of lineage-specific features, such as the presence of TF families specific to the green lineage in *Porphyridium purpureum*, haptophytes and stramenopiles. Our comprehensive pipeline also allowed us to identify fungal and cyanobacterial TF families in the algal nuclear genomes.

**Conclusions:**

This study provides examples illustrating the complex evolutionary history of algae, some of which support the involvement of a green alga in haptophyte and stramenopile evolution.

**Electronic supplementary material:**

The online version of this article (doi:10.1186/s12864-016-2610-9) contains supplementary material, which is available to authorized users.

## Background

In every living organism, developmental, morphological and physiological mechanisms, such as those allowing acclimation to environmental changes, are the result of genome expression modulation. One level of this modulation is related to gene expression, in which transcription factors are among the key players [1]. These regulators can be divided into two groups: transcription factors (TFs) and transcriptional regulators (TRs). These groups interact with each other and affect gene transcription. TFs are characterized by a DNA binding domain (DBD), an oligomerization domain (allowing interaction with other TFs, as well as with other transcriptional regulators) and a transcription regulation domain (allowing control of gene expression). These proteins (also called *trans*-factors) control the expression of multiple target genes by binding to specific DNA motifs in their promotor regions. TRs interact with TFs or with chromatin allowing genes to be transcribed either (1) facilitating the recruitment of the basal transcription machinery, or (2) modifying chromatin structure, making genes more accessible [2].

TFs are classified according to their DBD [3]. Most TFs have only one DBD, which can be present in one or multiple copies in the same sequence. However, some TFs can have several DBD types in their sequence [4].

Since the first study on the identification of TFs in four archaeal genomes [5], the increase in the number of sequenced genomes facilitates putative TF identification in unrelated taxa through *in silico* studies [6–10]. Such taxonomically diverse data allows comparative analyses between different species or lineages [6, 7, 9–13] and understanding of the evolutionary aspects through TFs [11, 14, 15]. This kind of study can reveal taxonomic characteristics (i.e., the specificity and expansion of TF families) of the TF complement of different organisms. *In silico* analysis of FTs performed on *Arabidopsis thaliana* (*A. thaliana*) showed that 45 % of TFs are plant specific. Moreover, a plant-specific expansion of the MYB superfamily was demonstrated (190 copies in the *A. thaliana* genome compared with 6 and 10 in *Drosophila melanogaster* and *Saccharomyces cerevisiae*, respectively) [6]. Another example of such lineage-specific expansion of a TF family is the retinoic acid receptors in the nematode *Caenorhabditis elegans*. Using the AnimalTFDB database, 239 putative TFs belonging to this family were identified, whereas in other animals, such as *Tetraodon nigroviridis,* this TF family is only represented by 19 members [10].

Among microalgae, TF complement comparative studies have been undertaken for stramenopiles [9] and to investigate the evolutionary history of both red and green algae among photosynthetic organisms [11, 15]. Microalgae arose from the endosymbiosis of a photosynthetic eukaryote, related to today’s cyanobacteria, by a primitive eukaryotic heterotroph. Glaucophyta, Rhodophyta and Chlorophyta all originated from this primary endosymbiosis [16, 17]. A series of secondary and tertiary endosymbioses would have then led to the diversity of microalgae observed today [18, 19]. Haptophytes would have appeared, as would stramenopiles, from the secondary endosymbiosis of both a green and a red alga by a heterotrophic eukaryote [19, 20]. Haptophytes are one of the key players in the evolutionary history of photosynthetic organisms [21] and are widely distributed among the photosynthetic unicellular eukaryotes in today’s oceans. However, *in silico* comparative studies in haptophytes are limited because few data are available.

Here, we conducted the first genome-wide identification and comparison of the TF complement in haptophytes using an optimized and automated pipeline. This analysis pipeline combines research for similarities with known TFs and protein domains using a large database containing plant, fungal, mammal and cyanobacterial TFs. Using our pipeline, we performed the *in silico* identification of the TF complement in three haptophytes (*Tisochrysis lutea*, *Emiliania huxleyi* and *Pavlova sp*) and two stramenopiles (the eustigmatophycea, *Nannochloropsis gaditana* and the diatom *Phaeodactylum tricornutum*), which are close organism groups [19, 22], as well as in the green alga *Chlamydomonas reinhardtii* and the red alga *Porphyridium purpureum*. We focused on the identification of the main families of TFs found in these microalgal species and compared their respective abundance in each. Moreover, the present study identified, for the first time, the presence of cyanobacterial TFs in each of the microalgal genomes studied.

## Results and discussion

### Evaluation of transcription factor identification accuracy

Pipeline analysis is essential for whole genome TF identification. Since no universal pipeline exists, each study uses its own. However, every pipeline is based on the same tools: a single identification with BLAST searches against a plant database [9, 15], and/or a single protein domain search with HMMER software focused on plant DBDs [11–13]. Several pipelines combine both methods so as to be more accurate and exhaustive [2, 8]. Moreover, the HMMER software is used either with the Pfam database or the combination of Pfam and another database. Our pipeline also combines the same identification strategies, but with some specificities: our analysis pipeline includes more protein domain databases (the eleven databases of the InterProScan consortium) and the research is not restricted to plants, but enlarged to fungi, algae and cyanobacteria.

**Fig. 1:**
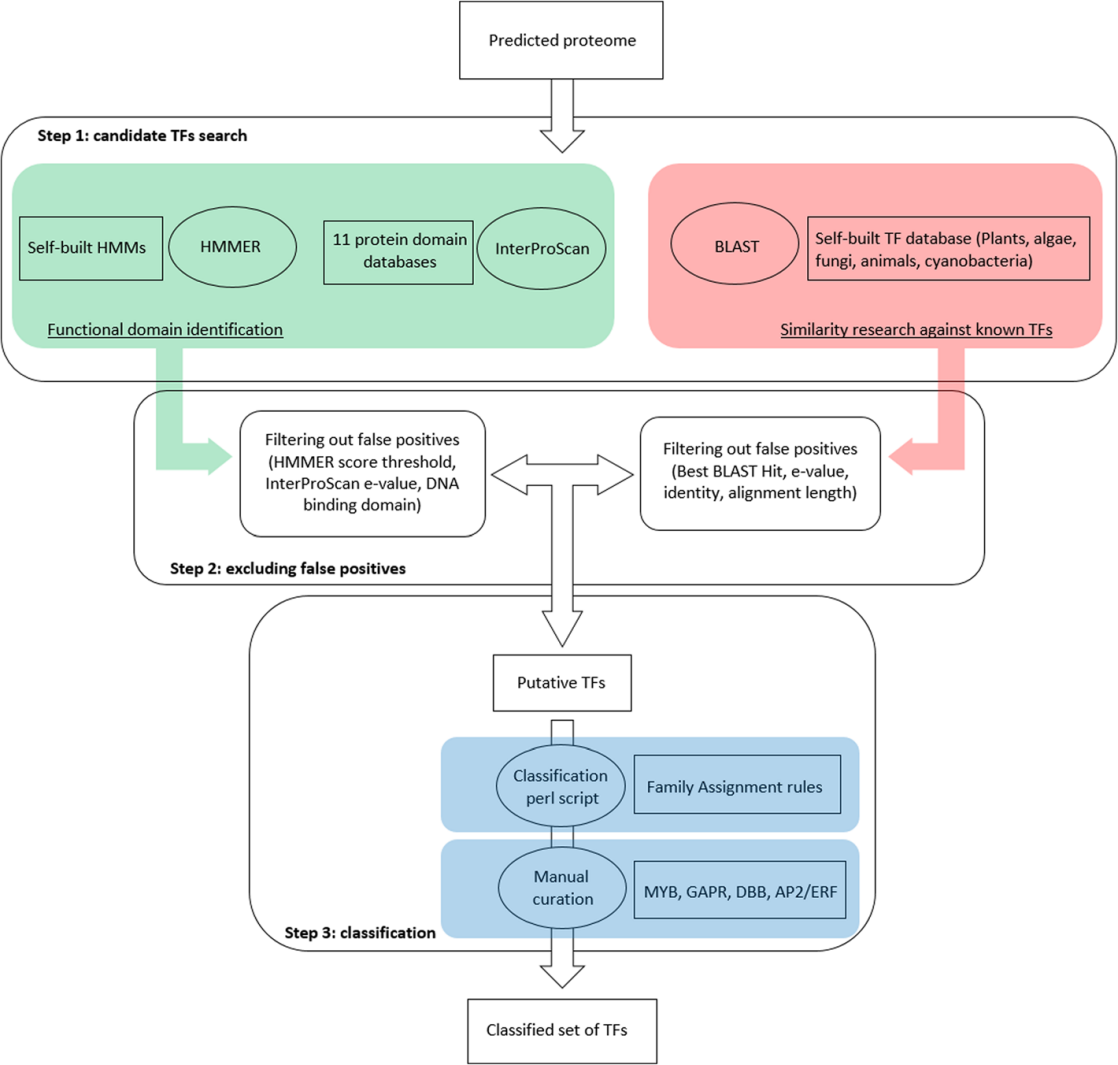
Identification pipeline. The pipeline is divided into three steps. Step One uses two strategies: i) a similarity search against an algae-based self-built database of known TFs with BLAST software; ii) functional domain annotation with InterProScan and HMMER software. The protein list obtained is the subject of the Step Two: the filtration of false positives according to specific parameters (see Methods). The last step consists in the classification of the putative TF list obtained in Step Two using a homemade perl script followed by manual curation for specific cases (see Methods)

In order to estimate the accuracy of our pipeline (Fig. 1), we applied it to the predicted proteome of *A. thaliana* and three cyanobacteria (see Methods section). The sensitivity and the PPV were measured in the same way as [23] and [24].

The analysis of the pipeline accuracy against eleven plant TF families showed that nine were identified with a good sensitivity and PPV values equal to one (Tables 1 and 2). Only, MADS and bHLH TF families were identified with a low sensitivity and a PPV value of 0.99, respectively. Using a more recent gold standard than [23] and [24], our sensitivity and PPV values are equivalent or better than previous pipelines [24, 25].

**Table 1:** Evaluation of the pipeline accuracy for each TF family for plant TFs. A sensitivity value less than one means inclusion of false negatives, and a PPV value less than one means inclusion of false positives

*A. thaliana*				
	This study		Riaño-Pachón et al., 2007 [24]	
TF family	sensitivity	PPV	sensitivity	PPV
AP2/ERF	169/169 = 1	169/169 = 1	0.99	1
ARF	37/37 = 1	37/37 = 1	0.91	0.95
bZIP	127/127 = 1	127/127 = 1	0.92	0.97
C2C2-Dof	47/47 = 1	47/47 = 1	0.97	0.97
C2C2-GATA	41/41 = 1	41/41 = 1	1	1
GARP	85/85 = 1	85/85 = 1	NA	NA
GRAS	37/37 = 1	37/37 = 1	0.97	0.97
MADS	145/146 = 0.99	145/145 = 1	0.92	0.95
NAC	138/138 = 1	138/138 = 1	1	0.99
WRKY	90/90 = 1	90/90 = 1	0.99	0.99
bHLH	225/225 = 1	224/225 = 0.99	0.80	0.92

**Table 2:** Evaluation of the pipeline accuracy for each TF family for cyanobacterial TFs. A sensitivity value less than one means inclusion of false negatives, and a PPV value less than one means inclusion of false positives

Cyanobacteria		
TF family	sensitivity	PPV
arsR	12/12 = 1	12/12 = 1
Bac_DNA_binding	6/6 = 1	6/6 = 1
BolA	3/3 = 1	3/3 = 1
Crp	15/15 = 1	15/17 = 0.88
FUR	9/9 = 1	9/9 = 1
GerE	34/34 = 1	34/34 = 1
GntR	5/5 = 1	5/6 = 0.83
LysR	15/15 = 1	15/15 = 1
SfsA	3/3 = 1	3/3 = 1

Concerning the cyanobacterial TF families, the sensitivity value was one for all families (no false negative identified). The PPV values were equal to one for cyanobacterial TFs, except for the GntR and Crp families (0.83 and 0.88, respectively). These lower PPV values are mostly due to the lower number of TFs in these organisms (i.e.*,* only one and two false positives for families GntR and Crp). These results indicate the high accuracy (low false positives identified) and performance (low false negatives) of our analysis pipeline for the *in silico* identification of TFs not only in plants and cyanobacteria but also for other organisms such as algae.

### Transcription factor content in algae

**Fig. 2:**
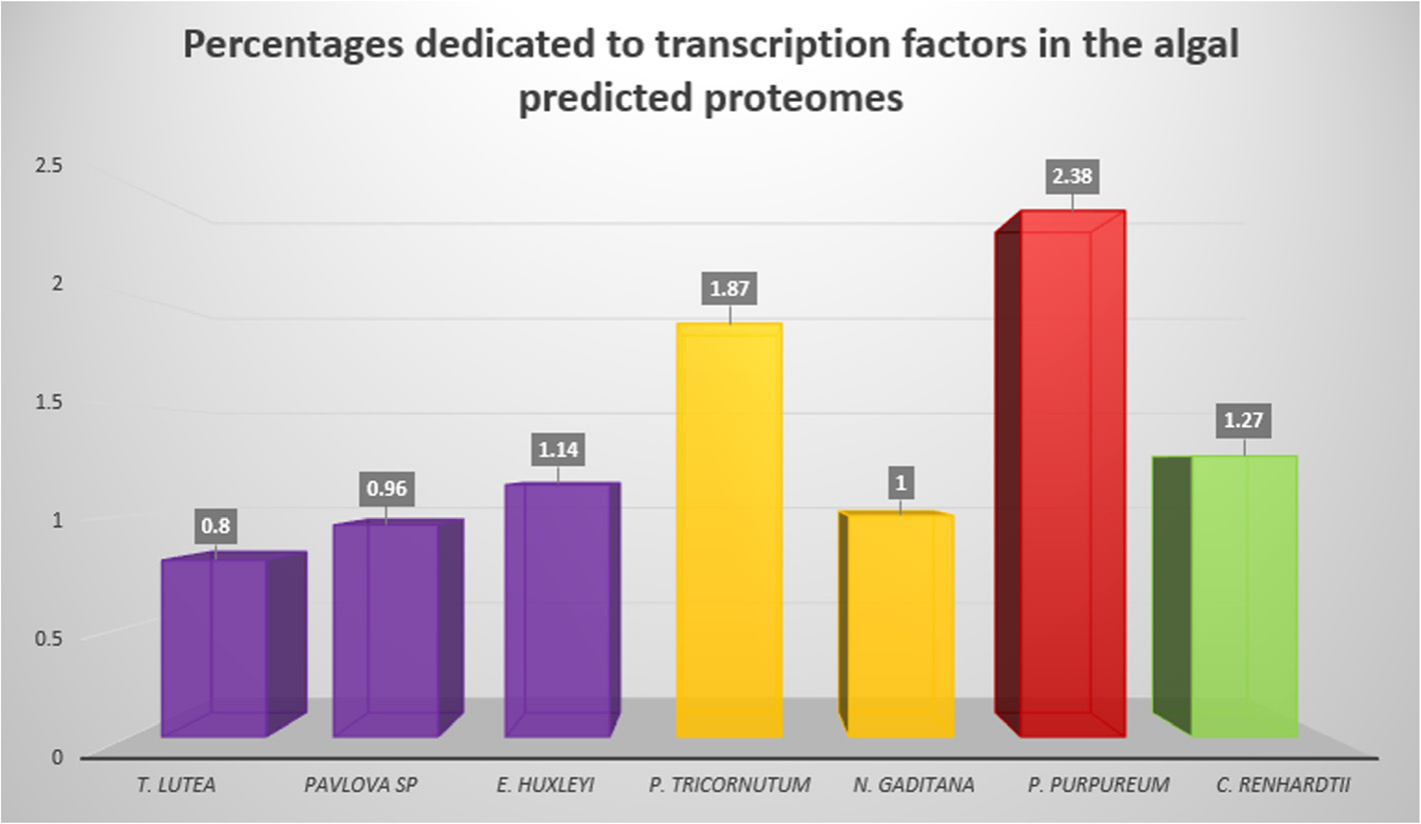
Percentages of the predicted proteomes dedicated to transcription factors in the 7 algae

In this study, predicted TFs from seven algae representing four different lineages were identified and classified using our analysis pipeline (Table 3). In total, 155,128 and 478 TFs were identified in the haptophytes *Tisochrysis lutea* (*T. lutea*), *Pavlova* sp. and *Emiliania huxleyi* (*E. huxleyi*), respectively. Concerning the two stramenopiles, 196 and 93 TFs were identified in *Phaeodactylum tricornutum* (*P. tricornutum*) and *Nannochloropsis gaditana* (*N. gaditana*), respectively. Finally, 199 and 212 TFs were identified in the rhodophyte *Porphyridium purpureum* (*P. purpureum*) and the chlorophyte *Chlamydomonas reinhardtii* (*C. reinhardtii*), respectively. All TFs identified belong to common families that are largely distributed between species studied. Here, the predicted TFs of the haptophytes *T. lutea*, *Pavlova* sp. and *E. huxleyi* were divided into 27, 24 and 25 families, respectively. Twenty-two families were reported for each of the stramenopiles (*P. tricornutum* and *N. gaditana*), while 25 and 37 families were identified for *P. purpureum* and *C. reinhardtii*. According to predicted proteomes, the proportion of TFs was estimated between 0.8 and 2.4 % (Fig. 2). Such percentages in microalgae are consistent with previous studies [9, 13]. By way of comparison across the eukaryotic world, the unicellular organism *Saccharomyces cerevisiae* dedicates 3.5 % of its proteome to TFs [26]; whereas the multicellular eukaryotes such as *Drosophilia melanogaster*, *A. thaliana* and *Homo sapiens*, contain 4.6, 5.9 and 8 to 9 % TFs, respectively [6, 26, 27]. In accordance with the fact that TFs play a role in morphology diversification of organisms [28–30] these proportions show a correlation between the complexity of organisms and the proportion of TFs found in the proteome of these organisms [2, 14, 31–33]. This is illustrated by the coincidence of TF families’ expansion with divergence of great eukaryotic lineages [11]. Indeed, it is well known that the evolutionary history of eukaryotes, especially plants, is punctuated by multiple biological processes, such as duplication [34–36] or domain shuffling, allowing modifications resulting in the emergence of new TF families [6, 11, 37]. These whole or partial genome duplications and domain shuffling have not been shown in algae. However, it can be reasonably assumed that such phenomena, leading to the emergence of new TF families, have also occurred in algae. This is suggested by the presence of TF families found only in green algae compared to the other algal lineages.

**Table 3:** Transcription factor families identified and their proportions in seven microalgae

TF family		*Tisochrysis lutea*	*Pavlova* sp	*Emiliania huxleyi*	*Phaeodactylum tricornutum*	*Nannochloropsis gaditana*	*Porphyridium purpureum*	*Chlamydomonas reinhardtii*
B3	ABI3/VP1	1 (0.65)	0 (0)	0 (0)	0 (0)	0 (0)	0 (0)	1 (0.47)
AP2/ERF	AP2	1 (0.65)	1 (0.78)	58 (12.13)	0 (0)	2 (2.15)	0 (0)	6 (2.83)
	ERF	1 (0.65)	6 (4.69)	99 (20.71)	2 (1.02)	2 (2.15)	0 (0)	9 (4.25)
bHLH		0 (0)	0 (0)	0 (0)	8 (4.08)	3 (3.23)	3 (1.51)	8 (3.77)
bZIP		3 (1.94)	3 (2.34)	6 (1.26)	25 (12.76)	11 (11.83)	21 (10.55)	20 (9.43)
C2C2	CO-like	0 (0)	0 (0)	0 (0)	0 (0)	0 (0)	0 (0)	1 (0.47)
	Dof	0 (0)	0 (0)	0 (0)	0 (0)	0 (0)	0 (0)	1 (0.47)
	GATA	5 (3.23)	1 (0.78)	4 (0.84)	0 (0)	0 (0)	2 (1.01)	12 (0.66)
	LSD	1 (0.65)	1 (0.78)	0 (0)	0 (0)	0 (0)	0 (0)	1 (0.47)
C2H2		8 (5.16)	8 (6.25)	37 (7.74)	4 (2.04)	5 (5.38)	60 (30.15)	5 (2.36)
C3H		13 (8.39)	7 (5.47)	47 (9.83)	11 (5.61)	5 (5.38)	8 (4.02)	22 (10.38)
CCAAT		3 (1.94)	0 (0)	2 (0.42)	3 (1.53)	3 (3.23)	3 (1.51)	1 (0.47)
CPP		1 (0.65)	0 (0)	4 (0.84)	5 (2.55)	1 (1.08)	2 (1.01)	3 (1.42)
CSD		3 (1,94)	4 (3.13)	25 (5.23)	5 (2.55)	1 (1.08)	3 (1.51)	2 (0.94)
DBB		0 (0)	0 (0)	0 (0)	0 (0)	0 (0)	1 (0.50)	0 (0)
E2F/DP		2 (1.29)	3 (2.34)	3 (0.63)	5 (2.55)	1 (1.08)	3 (1.51)	3 (1.42)
Fungal TRF		14 (9.03)	8 (6.25)	27 (5.65)	1 (0.51)	10 (10.75)	0 (0)	0 (0)
GARP	G2-like	4 (2.58)	4 (3.13)	5 (1.05)	2 (1.02)	0 (0)	2 (1.01)	4 (1.89)
	ARR-B	0 (0)	0 (0)	0 (0)	0 (0)	0 (0)	0 (0)	1 (0.47)
Homeobox	HB-other	16 (10.32)	14 (10.94)	28 (5.86)	0 (0)	0 (0)	2 (1.01)	1 (0.47)
	TALE	1 (0.65)	1 (0.78)	0 (0)	4 (2.04)	0 (0)	9 (4.52)	3 (1.42)
HSF		9 (5.81)	8 (6.25)	8 (1.67)	67 (34.18)	4 (4.30)	1 (0.50)	2 (0.94)
LIM		2 (1.29)	3 (2.34)	11 (2.30)	0 (0)	0 (0)	2 (1.01)	1 (0.47)
MADS-box	M-type	3 (1.94)	1 (0.78)	1 (0.21)	0 (0)	0 (0)	2 (1.01)	2 (0.94)
mTERF		5 (3.23)	0 (0)	6 (1.26)	5 (2.55)	2 (2.15)	5 (2.51)	4 (1.89)
MYB	MYB (3R)	1 (0.65)	0 (0)	3 (0.63)	2 (1.02)	5 (5.38)	1 (0.50)	1 (0.47)
	MYB (2R)	25 (16.13)	20 (15.63)	39 (8.16)	11 (5.61)	8 (8.60)	23 (11.56)	10 (4.72)
	MYB-rel	21 (13.55)	15 (11.90)	51 (10.69)	7 (3.57)	7 (7.53)	7 (3.52)	18 (8.65)
	MYB-SHAQKYF	1 (0.65)	2 (1.56)	1 (0.21)	7 (3.57)	8 (8.60)	16 (8.04)	4 (1.89)
NF-X1		0 (0)	0 (0)	0 (0)	0 (0)	0 (0)	0 (0)	1 (0.47)
NF-Y	NF-YA	0 (0)	1 (0.78)	1 (0.21)	1 (0.51)	1 (1.08)	1 (0.50)	0 (0)
	NF-YB	1 (0.65)	1 (0.78)	4 (0.84)	2 (1.02)	2 (2.15)	3 (1.51)	3 (1.42)
	NF-YC	3 (1.94)	4 (3.13)	1 (0.21)	8 (4.08)	6 (6.45)	6 (3.02)	2 (0.94)
Nin-like		0 (0)	1 (0.78)	0 (0)	0 (0)	1 (1.08)	4 (2.01)	15 (7.08)
S1Fa-like		0 (0)	0 (0)	0 (0)	0 (0)	0 (0)	0 (0)	1 (0.47)
SBP		0 (0)	0 (0)	0 (0)	0 (0)	0 (0)	0 (0)	23 (10.85)
Sigma-70		4 (2.58)	4 (3.13)	2 (0.42)	8 (4.08)	4 (4.30)	8 (4.02)	1 (0.47)
TUB		3 (1.94)	7 (5.47)	5 (1.05)	3 (1.53)	1 (1.08)	0 (0)	6 (2.83)
VARL		0 (0)	0 (0)	0 (0)	0 (0)	0 (0)	0 (0)	12 (5.66)
Whirly		0 (0)	0 (0)	0 (0)	0 (0)	0 (0)	0 (0)	1 (0.47)
WRKY		0 (0)	0 (0)	0 (0)	0 (0)	0 (0)	0 (0)	1 (0.47)
	Total	155	128	478	196	93	199	212

*ERF* Ethylene Response Factor, *bHLH* basic helix-loop-helix, *bZIP* basic leucine zipper, *CSD* Cold Shock Domain, *DBB* Double B-box, *TRF* Transcriptional Regulatory Factor, *HSF* Heat Shock Factor, *mTERF* mitochondrial transcription termination factor, *SBP* SQUAMOSA promotor binding protein, *VARL* Volvocine Algal RegA Like. Numbers in parentheses correspond to percentage of each family for each species. For the total number of TFs, number in parentheses corresponds to percentage of the predicted proteome dedicated to TFs

**Fig. 3:**
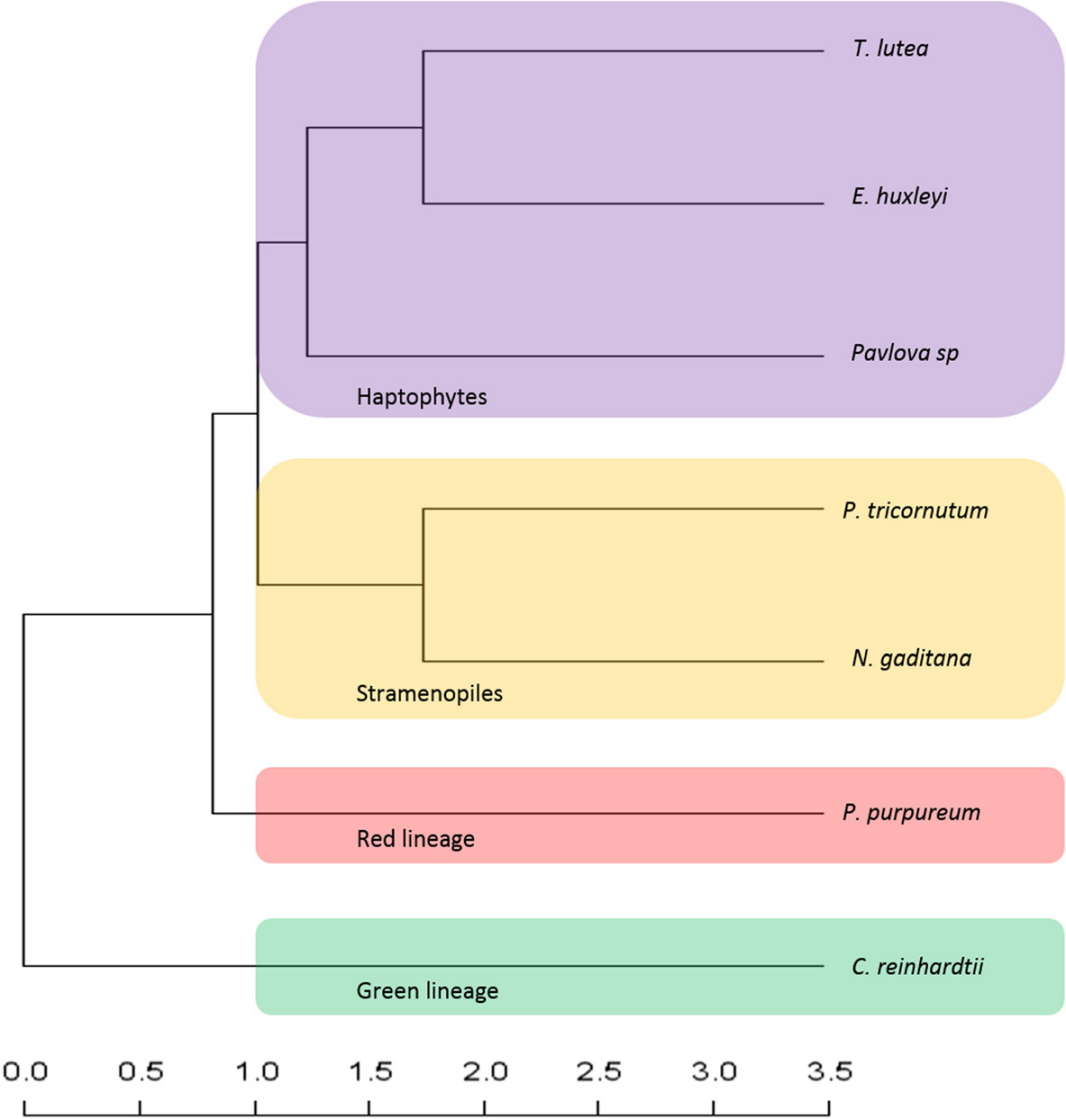
Dendrogram representing the repartition of the four lineages according to the presence/absence of TF families. The green lineage is colored in green, stramenopiles in orange, red lineage in red and haptophytes in purple. The scale indicates distance measurement

**Fig. 4:**
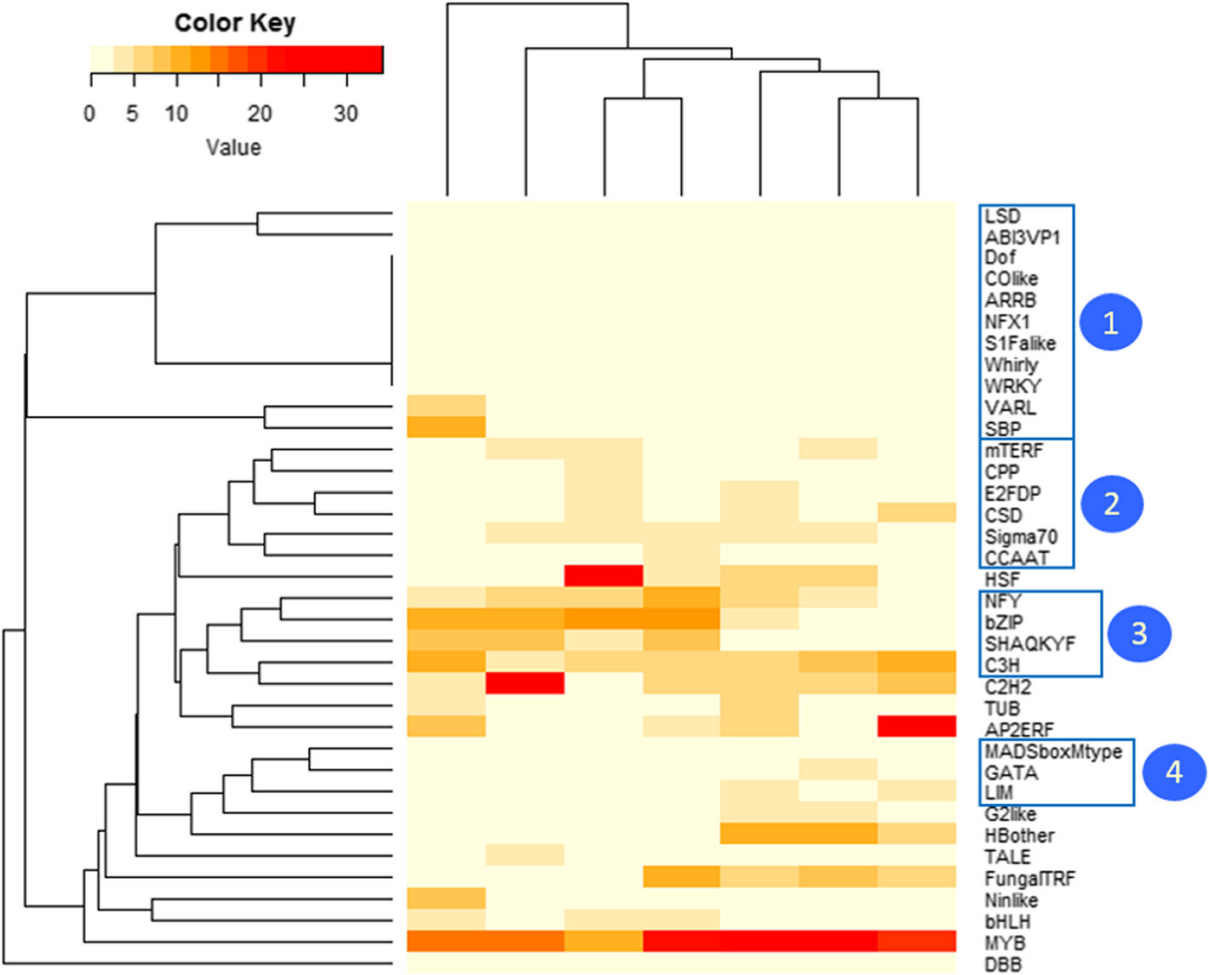
Heatmap showing the clustering of TF families according to their proportion in the algal genomes. Cluster 1 comprises TF families described as specific to the green lineage. Cluster 2 is composed of families with equivalent proportions across algal genomes. Cluster 3 is composed of families present in the 7 algae but in different proportions. Cluster 4 is composed of 3 families that are absent in stramenopiles

These lineage-specific gains and losses of TF families are a kind of mirror of their evolutionary history. To illustrate this idea, a binary table representing the presence/absence of TF families in seven algae representing four different lineages was performed. On this basis, a similarity matrix was computed to infer a dendrogram using R version 3.1.0 (Fig. 3). The resultant dendrogram (deposited in TreeBase: http://purl.org/phylo/treebase/phylows/study/TB2:S19079) confirms the relationship between algae derived from the four different lineages. Haptophytes, stramenopiles, red algae and green algae are clearly separated. We also found that *T. lutea* is more related to *E. huxleyi* than *Pavlova sp.,* as has been described in the literature [38, 39]. The rhodophyte *P. purpureum* is located between haptophytes and stramenopiles. This position is mostly due to the absence of MADS-box and C2C2-GATA families in stramenopiles, which makes them a more distant group from the four previous algae. Finally, the chlorophyte *C. reinhardtii* is the most distant from the others because of the presence of the TF families specific to the green lineage. This illustrates that the composition of this TF content is partly lineage specific. To discriminate the TF families, a haetmap was built using the data of Table 3. TF families were clustered according to their given proportions in the seven algal genomes (Fig. 4). Four interesting clusters were found: (i) TF families described as specific to green lineage. (ii) TF families with equivalent proportions among the 7 algal genomes. (iii) TF families present in the 7 algae but with different proportions. (iv) Finally, TF families only absent in stramenopiles.

In the following section, the TF content of the seven algae and their specificities of lineage, based on Table 3 and Fig. 4, are examined in more detail.

### Comparison of TF families among microalgae lineages

#### Common TF families with equivalent proportions

The proportions of each TF family in the seven algae were compared. We found that four families were present in similar proportions throughout the algal lineage (Table 3). Among these, the Cold Shock Domain (CSD) family is distributed around 1 to 5 % in analyzed algae. Our analysis pipeline identified for the first time three CSD TFs in the rhodophyte *P. purpureum,* representing 1.5 % of the predicted proteome. Moreover, this family was previously described as absent from red microalgae [15]. The absence of identification of CSD TFs from the red lineage may be explained by the fact that research on red microalgae was performed only in the genome of the extremophiles *Galderia sulfuraria* (*G. sulfuraria*) and *Cyanidioschyzon merolae* (*C. merolae)*. These organisms are adapted to the particular selection pressure due to their living environment (in hot springs such as in Yellowstone National Park) [40]. Consequently, the absence of this TF family from *G. sulfuraria* and *C. merolae* cannot be taken as a common characteristic of the red lineage.

The E2F/DP family, present in all eukaryotes and known for its involvement in the cell cycle [41], is also equally distributed among algae (around 1 to 3 %).

The MYB family is large, functionally diverse and represented in all eukaryote, such as algae (around 30 %). MYB factors are characterized by a highly conserved DNA-binding domain: the MYB domain. MYB TFs can be divided into different classes depending on the number of adjacent repeats. Three repeats of MYB protein are referred to as R1, R2 or R3, and repeats identified on other related MYB proteins are named in accordance with their similarity with R1, R2 and R3. Although most of these TFs are not functionally characterized in plants, some have been identified as involved in key mechanisms, such as cellular morphogenesis, secondary metabolism, response to biotic and abiotic stresses and signal transduction [42–45]. Finally, the last family equally distributed among algae is the Sigma-70 family. Members of the Sigma-70 family of sigma factors serve as components of the RNA polymerase that direct it to specific promoter elements. In photosynthetic eukaryotes, these Sigma-70 TFs are nuclear encoded and play a role in plastid transcription [46].

#### Common TF families with different proportions

Four cases of TF families exhibit a difference of proportion between species and are grouped in the cluster number 3 in the Fig. 4. Among these, the C3H type zinc finger family, whose DBD forms a zinc finger, is twice as common in haptophytes and green algae (around 10 %, except for *Pavlova* sp. (5.5 %)) as in stramenopiles and red algae (around 5 %) (Table 3). This protein family is widespread in the tree of life [47–49] and involved in the response to biotic and abiotic stresses [50, 51]. The second family that shows different proportions is the basic leucin-zipper (bZIP) TF family, which accounts for about 2 % in the three haptophytes analyzed in this study, while its proportion is about 10 % in the other algae (*P. tricornutum*: 12.8 %, *N. gaditana*: 11.8 %, *P. purpureum*: 10.6 % and *C. reinhardtii*: 9.4 %).

**Fig. 5:**
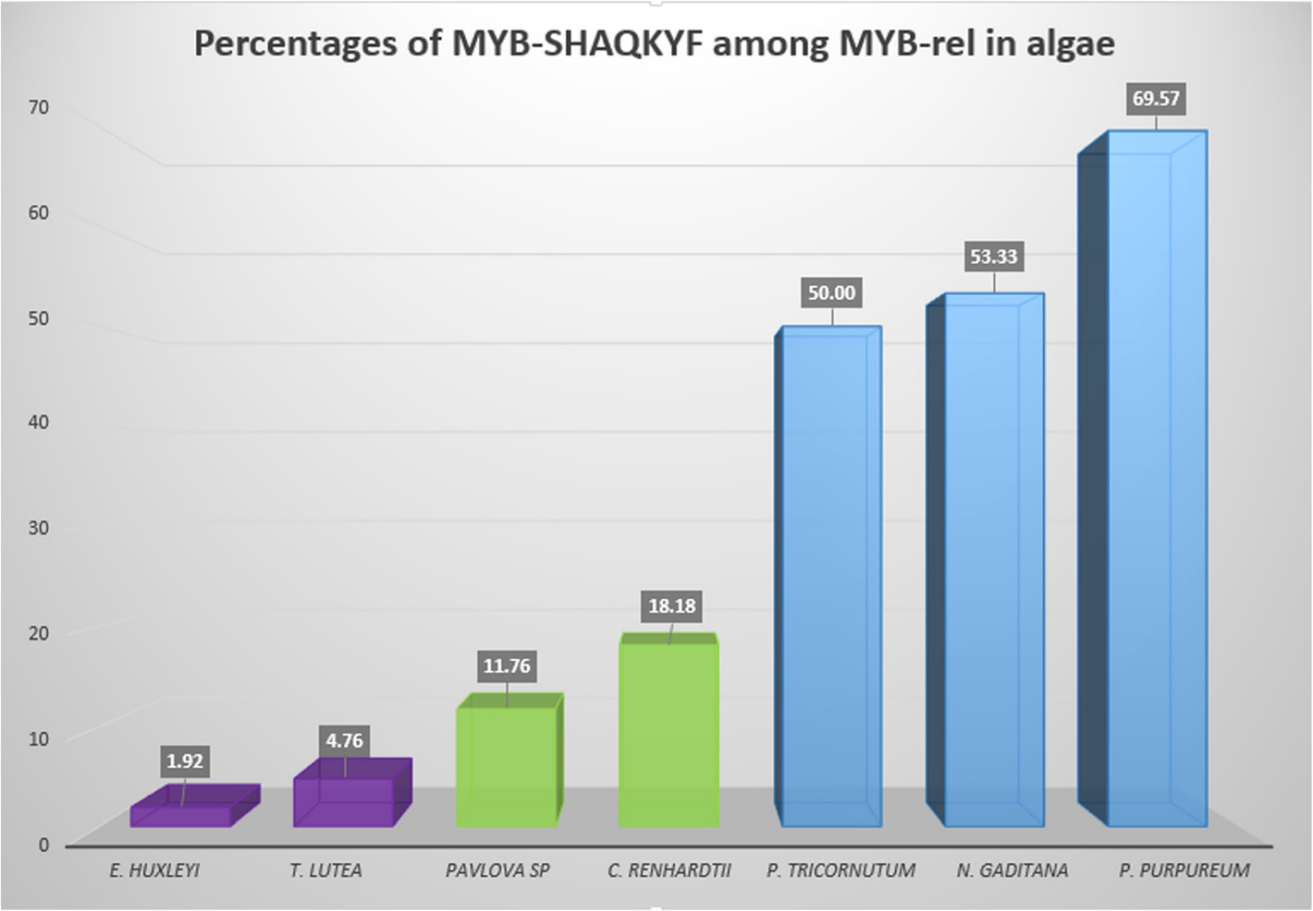
Percentages of MYB-SHAQKYF among MYB-related TFs in algae

The third case is that of a particular class of MYB-related TFs: the SHAQKYF-like TFs. This family was described in plants, green algae, as well as in stramenopiles and Amoebozoa [9, 52, 53]. MYB-SHAQKYF is a minority among MYB-rel in *E. huxleyi* and *T. lutea* (2 and 4.7 %, respectively). For *Pavlova* sp. and *C. reinhardtii,* non-negligible amounts of MYB-SHAQKYF were identified among MYB-rel (13.3 and 22.2 %, respectively). In contrast, MYB-SHAQKYF represent almost half of the MYB-rel TFs in the two stramenopiles *P. tricornutum* and *N. gaditana,* as well as in the rhodophyte *P. purpureum* (50, 53.3 and 69.6 %, respectively) (Fig. 5). Such a distribution, together with the presence of such TFs in Amoebozoa, suggests that MYB-SHAQKYF proteins have an ancient origin.

Finally, the Nuclear Factor-Y (NF-Y) family, also present in all eukaryotes is divided into three subunits: NF-YA, NF-YB and NF-YC. In plants, three subunits were identified [13]; these TRs are involved in mechanisms as diverse as chloroplast biogenesis, stress response, nodule formation, flowering time control, fatty acid biosynthesis, or response to absisc acid and blue light [54–59]. Subunits NF-YB and NF-YC form a dimer in the cytosol, which is then translocated into the nucleus. The NF-YB/NF-YC dimer interacts in the nucleus with the NF-YA subunit. The functional trimer binds to a *cis*-element called CCAAT-box in the promoter of its target genes [60, 61]. However, no NF-YA subunit was identified in *T. lutea* and *C. reinhardtii*. Such an absence in chlorophyte was previously reported using a similar approach for *C. reinhardtii*, *Volvox carteri* and *Ostreococcus tauri* [13]. The absence of the NF-YA subunit would therefore imply that it is impossible to form the functional trimer. However, it was demonstrated that other TFs are able to interact with the NF-YB and NF-YC subunits. For example, the NF-YB/NF-YC complex can interact with a TF belonging to the C2C2-CO-like family thanks to its CCT domain [62]. Moreover, the interaction between the NF-YB/NF-YC complex and bZIP TFs of *A. thaliana* is sufficient to activate the transcription of target genes, either in the presence or absence of abscisic acid (ABA) [63]. Alternatively, the NF-YB/NF-YC dimer could be active without NF-YA in these taxa.

#### TF family expansion

During evolutionary history, duplication events occur. Following these duplications, the number of genes of a given family increases. These gene family expansions may be lineage or species specific [64]. Contrary to the other algae in which the MYB family is the most represented, in *P. tricornutum*, *E. huxleyi* and *P. purpureum,* another TF family is more represented because of the expansion phenomenon. In the stramenopile *P. tricornutum*, the Heat Shock Factor family (HSF) was the most represented among the TF families (34.2 % of the TF content) (Table 3). Such a proportion of HSF was previously shown in the diatoms *P. tricornutum*, *T. pseudonana* and *Fistulifera solaris* [9, 65]. This expansion seems to be specific to diatoms since neither *N. gaditana* nor other photosynthetic stramenopiles exhibit such expansion of HSFs [9].

In the haptophyte *E. huxleyi*, the most represented family, accounting for 33 %, is AP2/ERF, involved in growth and development as well as various responses to environmental stimuli. This family was described as specific to the green lineage [15] and its expansion in *E. huxleyi* was also previously described [9]. However, such a proportion of the AP2/ERF is not common to all haptophytes since *T. lutea* and *Pavlova sp*. have AP2/ERF proportions of 1.8 and 5.5 %, respectively, which are close to values recovered for stramenopiles and green algae, respectively. The non-detection of the AP2/ERF family in the Rhodophyta *P. purpureum* is noteworthy, confirming the absence of AP2/ERF in algae belonging to the red lineage [12, 15].

Finally, the C2H2 type zinc finger family was identified as the most represented family in the rhodophyte *P. purpureum*. We found that the C2H2 proportion represents 30.2 % compared to less than 8 % in the other algae. Interestingly, in the two extremophiles, *G. sulfuraria* and *C. merolae*, the C2H2 family was reported to account for less than 5 % [12].

These examples of lineage or species-specific TF expansion illustrate the phenomena that govern the story of TF evolution: gene duplication [66] and diversification through the emergence of lineage-specific families via functional domain shuffling [4, 6, 14, 67]. In the algal world, one of the best examples of lineage-specific TF families is the “green TFs family”, which are specific to the green lineage.

#### Lineage-specific TF families

##### Are TF families specific to the green lineage highly specific?

Previous comparative studies of the TF content of diverse photosynthetic organisms reveal that some TF families are specific to the green lineage because of their absence from red microalgae [11, 15]. Among all green lineage-specific TF families identified in this study, only nine families were present in the green algae *C. reinhardtti*: NF-X1, S1Fa-like, SBP, VARL, Whirly, WRKY, GARP-ARR-B, C2C2-CO-like and C2C2-Dof (Table 3). However, some TF families previously described as specific to the green lineage were also identified in haptophytes, stramenopiles or in the rhodophyte *P. purpureum*. First of all, one TF belonging to the ABI3/VP1 family was identified in *T. lutea* and the C2C2-LSD family have one member in both *T. lutea* and *Pavlova sp*. In the heatmap (Fig. 4), these two TF families are clustered with the nine families only identified in *C. reinhardtii*. Moreover, the CSD family was identified in all predicted proteomes and the AP2/ERF and TUB families are absent in *P. purpureum,* but present in the six other algae. Another interesting finding is the unique identification of a member of the Double B-box (DBB) family in *P. purpureum*. This family had only previously been identified in land plants [68] and was thought to be involved in light signal transduction mechanisms, such as early photomorphogenic development of *A. thaliana* [69–72].

This presence of “green TFs” in algae that do not belong to the green lineage could be explained either (i) by a loss of these families during evolutionary history of rhodophytes, or (ii) by the acquisition of these families by horizontal gene transfer from a green algal endosymbiont to the nuclear genome. This last hypothesis is consistent with the endosymbiosis of a green and a red alga in the evolutionary history of haptophytes and stramenopiles [19].

##### Specific features of stramenopiles

The stramenopiles *P. tricornutum* and *N. gaditana* are distinguished by the absence of the C2C2-GATA family and the MADS-box family, which are involved in plant homeotic functions [73–75] (Table 3). These results confirm those of Rayko et al. [9] for stramenopile micro- and macro-algae. Moreover, our results also highlight the absence of TFs from the LIM family in stramenopiles, while LIM TFs are present in all other studied algae. LIM, C2C2-GATA and MADS-box families are clustered together in Fig. 4. To examine whether these features are shared by other stramenopiles not investigated in this work, a specific research of LIM, MADS-box and C2C2-GATA TFs was carried out in the two diatoms *Pseudo-nitzschia multiseries* and *Fragilariopsis cylindrus*. No member of these families was identified (data not shown). By contrast, the MADS-box, C2C2-GATA and LIM families were identified in *P. purpurem* and *C. reinhardtii* (this study), as well as in other chlorophytes and rhodophytes (the green algae *Bathycoccus prasinos*, *Micromonas pusilla*, *Micromonas sp*, *Ostreococcus lucimarinus*, *Ostreococcus sp*, *Ostreococcus tauri* and *Volvox carteri*; the red algae *C. merolae* and *G. sulfuraria*) [12, 13]. This repartition suggests that the MADS-box, C2C2-GATA and LIM families were present in the hypothetical ancestor of the algae and secondarily lost in stramenopiles.

Another feature of stramenopiles concerns some particular combinations of functional domains. Two domain associations shared by both stramenopiles *N. gaditana* and *P. tricornutum* were identified. The first is composed of a bHLH domain and a PAS domain (named after the three first sequences in which it was identified (Per, Arnt, Sim)) and the second by a bZIP and LOV (Light, Oxygen, Voltage) domain combination. The bHLH-PAS TFs are well known in vertebrate TFs in which two PAS domains are present, contrary to the stramenopile sequences that have only one PAS [9, 76]. In vertebrates, the PAS domains are involved in the dimerization of PAS domains containing TFs, such as the Hypoxia Inducible Factor [77, 78]. The presence of bHLH and PAS domains in the same sequence in both vertebrates and stramenopiles may be an example of convergent evolution, which suggests that this fusion occurred in a parallel fashion in different lineages.

The second stramenopile specific combination is that of the bZIP and LOV domains. These sequences, called aureochromes, are an atypical case that couple both blue light receptor and transcription factor functions [79]. We identified three and four aureochromes in *N. gaditana* and in *P. tricornutum*, respectively. Such sequences have only been identified in photosynthetic stramenopiles [9, 79–82]. In marine environments, the sea water absorbs wavelengths other than blue, which are the only wavelengths to travel long distances within the water column [83]. Blue light is thus expected to play an important role in algae, as suggested by the involvement of aureochromes in key mechanisms such as the cell cycle [84]. Moreover, mechanisms like photomorphogenesis and phototropism observed in algae [85] are influenced in land plants by phototropins [86]. These are blue light receptors harboring two LOV domains and have a role in signal transduction [87]. Thus, aureochromes are lineage-specific TFs evolved by photosynthetic stramenopiles that confer an adaptive capacity for success in an aquatic environment.

##### Specific features of haptophytes

The bHLH TFs were identified in the predicted proteome of *P. tricornutum*, *N. gaditana*, *C. reinhardtii* and *P. purpureum,* but not in the three haptophytes (Table 3). Nevertheless, bHLH is one of the most widespread TF families in eukaryotes and the second most represented in plants [13, 88]. This repartition suggests that the bHLH TF family was secondarily lost in *T. lutea*, *E. huxleyi* and *Pavlova* sp. These results confirm previous conclusions derived from the comparison of the TF content composition of six stramenopiles with *E. huxleyi* [9], and extends the number of haptophyte organisms sharing this common absence of bHLH families.

Interestingly, we identified two and four Heat Shock transcription factors (HSFs) in *E. huxleyi* and *T. lutea*, respectively, that share the association of a HSF DBD with a PAS domain. Moreover, two other HSF proteins, harboring two PAS domains, were identified only in *T. lutea*.

The HSF domain is known for playing a role in stress perception in all categories of living organisms [89]. Its sensor function is applied to stimuli such as light, oxygen or redox potential. Such stimuli are also known to induce HSF expression. In plants in particular, HSFs are involved in response to oxidative stress and redox stat changes [90, 91]. This functional convergence led us to hypothesize that the sensor function of the PAS domain may play a role in the detection of stimuli involved in HSF activation. The PAS domain also enables protein-protein interactions, especially with other PAS-containing proteins [89, 92]. This function may stabilize the homotrimer formed by activated HSFs. Likewise, four TFs have the undescribed association of a PAS domain and a homeobox domain in *T. lutea*.

### Potential gene transfer cases

#### Identification of cyanobacterial TFs in the nuclear genome of algae

Remarkably, our TFs prediction pipeline allowed the identification of cyanobacterial TFs in the predicted proteome of all the microalgae studied (Table 4). We investigated whether the presence of these genes could be due to bacterial contamination, and if not, whether these genes are localized in the nuclear, chloroplastic or mitochondrial genome. Because information concerning bacterial contamination are only available for *T. lutea* (G. Carrier, pers. Com.), *Pavlova* sp. (transcriptomic data) and *C. reinhardtii* (JGI portal), it only was possible to answer the contamination question for these three algae. It allowed us to conclude that *T. lutea*, *Pavlova* sp. and *C. reinhardtii* cyanobacterial TFs identification are not due to bacterial contamination. Concerning the localization of the cyanobacterial TFs in the algae, we cannot draw any conclusions for *Pavlova* sp., for which no mitochondrial or chloroplastic genome are available. For *P. purpureum* the TFs are not localized in the chloroplastic genome; however, since the mitochondrial genome is not available, we cannot make a conclusion about a mitochondrial localization. We found that these TFs are nuclear genes for *T. lutea*, *E. huxleyi*, *P. tricornutum*, *N. gaditana* and *C. reinhardtii*.

**Table 4:** Number of cyanobacterial transcription factors (TFs) identified in the seven algae for each TF family

TF family	*T. lutea*	*Pavlova* sp	*E. huxleyi*	*P. tricornutum*	*N. gaditana*	*P. purpureum*	*C. reinhardtii*
arsR	0	0	0	0	1	0	0
Bac_DNA_binding	1	1	1	1	1	0	1
BolA	2	2	7	4	4	3	5
GerE	1	0	2	2	1	0	0
LysR	0	0	0	0	1	0	0
SfsA	1	3	2	0	1	2	2

BolA TFs were previously identified in the chlorophyte *C. reinhardtii*, the rhodophyte *Cyanidoschyzon merolae*, the diatom *Thalassiosira pseudonana* and the cryptophyte *Guillardia theta* [24]

Only one TF belonging to the arsenic resistance operon regulator (arsR) family was identified, in *N. gaditana*. This family is involved in stress response to metal ions in cyanobacteria [93]. Considering the Bac_DNA_Binding family, one member was identified in all the algae except in *P. purpureum*. This protein family is involved in transcription regulation, transposition and DNA chaperones [94, 95]. Several members of the BolA family were identified in all algae. BolA is a widespread family identified in all groups of the tree of life [2] and is involved in cell cycle regulation and abiotic stress response in cyanobacteria [96]. The GerE family which is part of a two component response regulator was only identified in haptophytes *T. lutea*, *E. huxleyi* (except for *Pavlova sp*.), and in the two stramenopiles *N. gaditana* and *P. tricornutum*. This family is characterized by the presence of a LuxR DBD and involved in processes such as signal transduction [97], quorum sensing [98] and sporulation [99]. One member of LysR protein was identified in *N. gaditana*. In cyanobacteria, this family is involved in CO_2_ fixation [100] and nitrate assimilation [101]. Finally, the SfsA family was identified in all algae except *P. tricornutum*. SfsA TF is known to be involved in sugar fermentation [102].

So far, no genome-wide TF identification study has shown the presence of such sequences in microalgae, except for the BolA family in the chlorophyte *C. reinhardtii*, the diatom *Thalassiosira pseudonana*, the rhodophyte *C. merolae* and the cryptophyte *Guillardia theta* [2]. Since these TF families are found either in cyanobacteria or bacteria, their presence in the algal genomes could be explained either by an endosymbiotic gene transfer (EGT), which is a gene transfer taking place from the chloroplastic genome to the nuclear genome during evolutionary history [103, 104], or a horizontal gene transfer (HGT) from a prokaryotic organism to the algal genome [105].

#### Fungal TRF: fungus in algae

The TF families described above are of bacterial type, but TFs from the fungal TRF family (also called Zn-clus) were also identified. These TFs are abundant and well described in fungi [106]. Their DBD is characterized by a conserved CysX2CysX6CysX5−16CysX2CysX6−8Cys motif. The six conserved cysteines coordinate two Zn(II) ions allowing correct folding of the domain [107]. This DBD was first identified in the *Saccharomyces cerevisiae* Gal4 TF [108]. Members of this TF family are implicated in the regulation of genes involved in diverse mechanisms, such as amino acid biosynthesis [109], multidrug resistance [110], ethanol catabolism [111] or lipid catabolism [112, 113].

Fungal TRF were identified in *T. lutea*, *Pavlova sp*., *E. huxleyi*, *N. gaditana* and *P. tricornutum*. However, no fungal TRF were identified in either *C. reinhardtii* or in *P. purpureum*. In previous studies TFs from this family were identified in the rhodophyte *G. sulfuraria* [12, 15].

This presence of fungal type TFs in algal genomes is another illustration of the complex evolutionary history of algae [114]. Multiple endosymbiosis resulting in the algal diversity [18] is punctuated by numerous gene transfer events. These gene transfer events comprise both EGT [115, 116], as the original case of HGT from bacteria to the plastid genome [117], or from bacteria or archaebacteria to the nuclear genome [40, 105, 118, 119]. In these HGT, the donor organism is prokaryotic, but interesting cases of HGT from a fungus to an alga were recently shown [120]. All these gene transfers give rise to metabolic and regulatory diversity, leading to adaptation of algae to a wide variety of environments and conditions.

## Conclusion

**Fig. 6:**
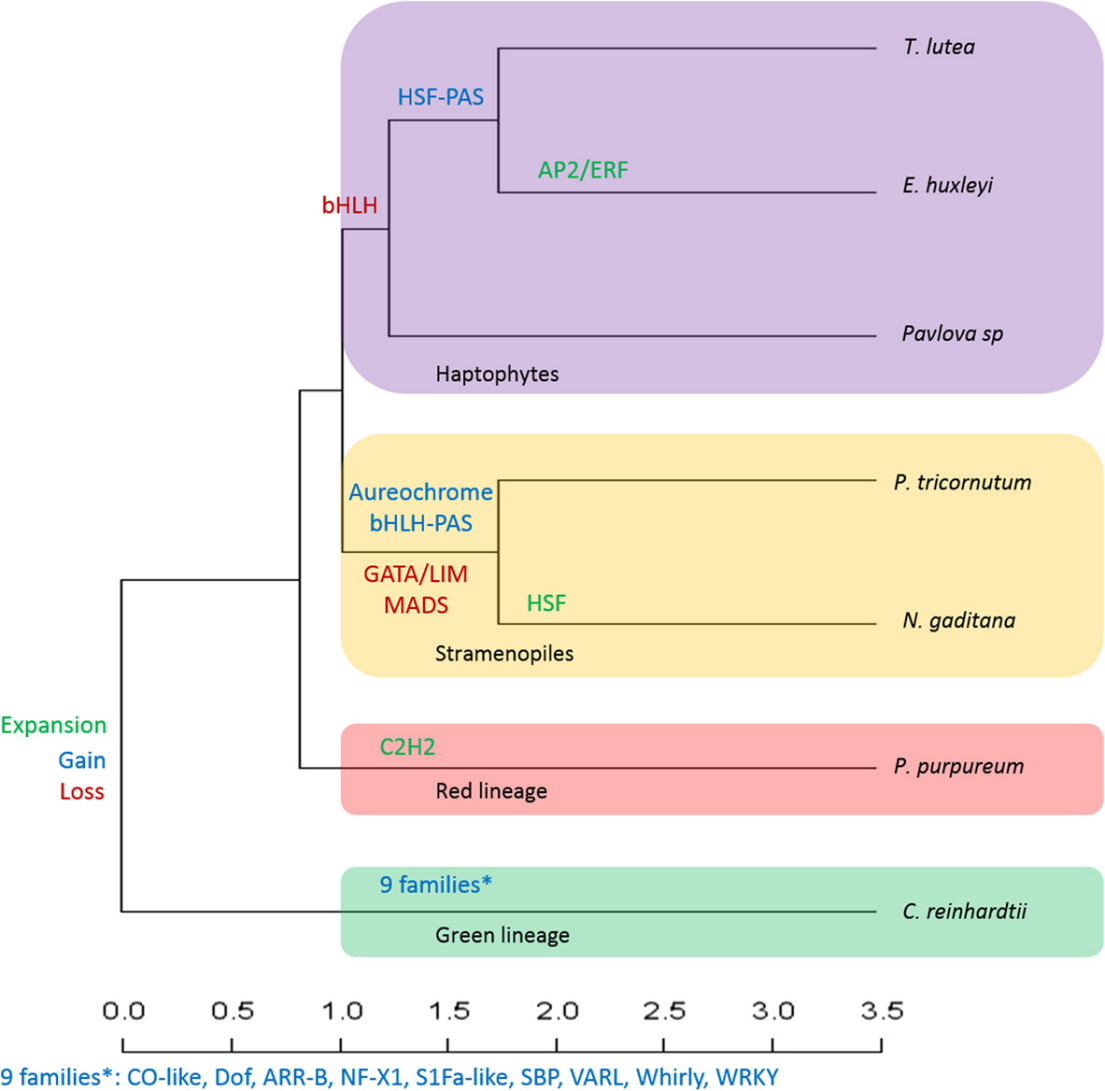
Expansion, gain and loss of TF families during the evolutionary history of microalgae

Using a pipeline with very good sensitivity and PPV for both plant and cyanobacterial TFs, we undertook the first genome-wide identification of TFs in haptophytes, coupled with a comparison of TF content between haptophytes and other algal lineages. The identification highlighted the presence of cyanobacterial TFs in algal nuclear genomes, which is likely to originate from either an EGT or an HGT. Moreover, members of the Fungal TRF family were identified in *T. lutea*, *Pavlov* asp, *E. huxleyi*, *P. tricornutum* and *N. gaditana*. The presence of fungal type TFs in algal genomes also illustrates the complex evolutionary history of these organisms. This comparison study confirms and extends lineage-specific features highlighted between haptophytes and stramenopiles by previous work [9] and extends the panel of genomes used for this comparison (Fig. 6). In order to investigate the evolutionary history of organisms and genome-wide studies, some gaps need to be filled and the red algae are one of them. In this kind of study, the only two red algae used are the two extremophiles *G. sulfuraria* and *C. merolae*. The extreme environmental pressures they face make these two algae peculiar cases that should not be considered representative of the red lineage. Here, we used mesophilic species *P. purpureum*. Availability of genomic data from haptophytes is also lacking. In this study, we provide the first genomic data of *T. lutea*. The characteristics revealed include some clues consistent with the hypothesis of an endosymbiosis of green and red algae in the evolutionary history of haptophytes and stramenopiles [19]. Therefore, this work provides a basis to better understand gene regulation in *T. lutea,* which is a species of ecological interest as part of haptophytes, a diverse and often ecologically dominant group in the planktonic photic realm [121].

## Methods

### Source datasets

The predicted proteomes used in this study were downloaded from different sources (Additional file 1: Table S1). The *C. reinhardtii* CC-503 cw92 mt+, *P. tricornutum* CCAP1055/1 and *E. huxleyi* CCMP1516 predicted proteomes were downloaded from the JGI genome portal at http://genome.jgi.doe.gov/. The *N. gaditana* CCMP526, *Pavlova sp.* CCMP459 and *P. purpureum* DBLAB2 predicted proteomes were downloaded from http://nannochloropsis.genomeprojectsolutions-databases.com/, http://data.imicrobe.us/project/view/104 and http://cyanophora.rutgers.edu/porphyridium/, respectively. The genome of the *T. lutea* CCAP927/14 strain was recently sequenced and annotated in our laboratory (data not shown). Raw read data are available at SRA (RUN: SRR3156597).

### Identification and classification of transcription factors

The TF identification and classification pipeline was calibrated with the model plant *A. Thaliana* (TAIR 10). Overall, the pipeline uses two strategies: (1) a similarity research with BLAST software against a self-built database of known TFs from algae, *A. thaliana*, *Saccharomyces cerevisiae* and cyanobacteria; (2) identification of TF DBDs with InterProScan and HMMER software. The compilation of software results allowed us to obtain a putative list of TFs (Fig. 1).

#### Construction of a TF database for BLAST software

The TF database is composed of TFs from different organisms (the model plant *A. thaliana*; the green algae *Bathycoccus prasinos*, *Chlorella sp*, *Coccomyxa sp*, *Micromonas pusilla*, *Micromonas sp*, *Ostreococcus lucimarinus*, *Ostreococcus sp*, *Ostreococcus tauri* and *Volvox carteri*; the red algae *Cyanidioschyzon merolae* and *Galdieria sulfuraria*; the diatom *Thalassiosira pseudonana* and the yeast *Saccharomyces cerevisiae*). These sequences were retrieved from online databases (Additional file 2: Table S2). Since algae originate from the engulfment of a cyanobacteria-like organism by a primitive eukaryotic heterotroph, we added all cyanobacterial TFs of the cTFbase [8] to the self-built database.

#### Identification of protein functional domains

Each protein domain contained in the protein domain databases is stored as a Hidden Markov Model (HMM) and linked to a putative function. This statistical method computes a matrix based on the multiple alignments of a protein domain [122]. For functional domain annotation of all the predicted proteomes, we employed InterProScan 5 version 5.4-47.0 [123], which uses a consortium of eleven protein domain databases (PROSITE, HAMP, Pfam, PRINTS, ProDom, SMART, TIGRFAMs, PIRSF, SUPERFAMILY, CATH-Gene3D and PANTHER). However, twelve DBDs (G2-like, BELL, HD-ZIP, HRT, NF-YB, NF-YC, SAP, STAT, Trihelix, VOZ, WOX and VARL) are not supported by the eleven databases of the consortium and were added through multiple alignments available in the TF databases PlantTFDB [13] and PlnTFDB [12] with HMMER3, v3.1b1 [124].

#### Pipeline description

##### First step

Sequences of each predicted proteome were analyzed in parallel by HMMER (*hmmscan*, default parameters), InterProScan (default parameters) for protein functional domains and by BLAST (*e-value* threshold 10^−10^) for a similarity search against known TFs (Fig. 1).

##### Second step

The results of each software analysis were filtered using different homemade PERL scripts. For InterProScan, false positives were filtered out to keep only annotated domains that had an *e-value* above or equal to 10^−3^. Among these, only TFs DBDs were conserved. For HMMER, filtration was done on the score value. Sequences with a significant *hmmscan* match (according to the database thresholds) were added as TF candidates. For BLAST searches, the filtering step was applied with an identity percentage threshold of 35 % and an alignment length threshold of 100 residues. Then, the best-BLAST hit was taken for each query. Finally, the results of all software processes were combined in one file.

##### Third step

Once identified, putative TFs were classified into specific families according to their DBD(s). We used a compilation of the “family assignment rules” described by the web databases PlantTFDB [13], PlnTFDB [12] and cTFbase [8], as well as previous studies [9, 11]. A PERL script was used to automatically classify the putative TFs in families following the assignment rules.

##### Final step

Manual curation was necessary, in particular for three complex cases: (1) MYB, where the calibration stage revealed that filtration of the *e-value* score generated false negatives. To overcome this, MYB identification was performed using the same protocol, with the exception of the validation step of the *e-value* scores on the InterProScan result. Moreover, each candidate was manually inspected (BLAST) to confirm each MYB domain and classify putative TFs in each family (MYB-3R, MYB-2R and MYB-related). (2) G2-like, due to the absence of a G2-like domain in the InterProScan database and its close similarity to the MYB-SHAQKYF domain, cross-annotation between these two domains was manually checked using HMMER. (3) TF families characterized by the repetition of a single domain; for proteins identified as belonging to the DBB and AP2/ERF families, the presence of two or more B-Box or AP2/ERF domains, respectively, was verified.

#### Evaluation of pipeline accuracy

To estimate the accuracy and reliability of our identification method, we applied our pipeline to the predicted proteome of *A. thaliana* (TAIR 10) and compared the identification of eleven well-annotated families to published datasets [13], used as a gold standard. For the identification of cyanobacterial TFs, we applied our pipeline to *Synechocystis* sp. PCC 6803 (GeneBank Assembly: GCA_000009725.1), *Synechococcus* sp. CC9605 (downloaded from cyanobase) and *Nostoc punctiforme* PCC73102 (GeneBank Assembly: GCA_000020025.1) predicted proteomes and compared our prediction results with published data [8]. The accuracy was evaluated by the measurement of sensitivity:

$$ \frac{True\  positives}{True\  positives+ False\  negatives} $$

and Positive Predictive Value (PPV):

$$ \frac{True\  positives}{True\  positives+ False\  positives} $$

A sensitivity value of less than one means inclusion of false negatives and a PPV of less than one means inclusion of false positives.

### Availability of data and material

The datasets supporting the conclusions of this article are included within the article (and its additional files).

## Additional files

Additional file 1: Table S1. Source datasets. Table listing the reference of the genomic data used in this study. (XLSX 11 kb) Additional file 2: Table S2. Sources for the self-build TF database. Table listing sources for the building of the self-build transcription factor database. (XLSX 11 kb)

## Abbreviations

DBD: DNA binding domain
EGT: endosymbiotic gene transfer
HGT: horizontal gene transfer
HMM: Hidden Markov Model
TFs: transcription factor
TRs: transcriptional regulators

## References

[1] Heydarizadeh P, Marchand J, Chenais B, Sabzalian MR, Zahedi M, Moreau B, Schoefs B (2014). Functional investigations in diatoms need more than a transcriptomic approach. Diatom Res.

[2] Richardt S, Lang D, Reski R, Frank W, Rensing SA (2007). PlanTAPDB, a phylogeny-based resource of plant transcription-associated proteins. Plant Physiol.

[3] Luscombe NM, Austin SE, Berman HM, Thornton JM (2000). An overview of the structures of protein-DNA complexes. Genome Biol.

[4] Charoensawan V, Wilson D, Teichmann SA (2010). Lineage-specific expansion of DNA-binding transcription factor families. Trends Genet.

[5] Aravind L, Koonin EV (1999). DNA-binding proteins and evolution of transcription regulation in the archaea. Nucleic Acids Res.

[6] Riechmann JL, Heard J, Martin G, Reuber L, Jiang C, Keddie J, Adam L, Pineda O, Ratcliffe OJ, Samaha RR, Creelman R, Pilgrim M, Broun P, Zhang JZ, Ghandehari D, Sherman BK, Yu G (2000). Arabidopsis transcription factors: genome-wide comparative analysis among eukaryotes. Science.

[7] Martínez-Bueno M, Molina-Henares AJ, Pareja E, Ramos JL, Tobes R (2004). BacTregulators: a database of transcriptional regulators in bacteria and archaea. Bioinforma Oxf Engl.

[8] Wu J, Zhao F, Wang S, Deng G, Wang J, Bai J, et al. cTFbase: a database for comparative genomics of transcription factors in cyanobacteria. BMC Genomics. 2007;8:104. 10.1186/1471-2164-8-104 PMC1858693 17439663

[9] Rayko E, Maumus F, Maheswari U, Jabbari K, Bowler C (2010). Transcription factor families inferred from genome sequences of photosynthetic stramenopiles. New Phytol.

[10] Zhang H-M, Chen H, Liu W, Liu H, Gong J, Wang H, et al. AnimalTFDB: a comprehensive animal transcription factor database. Nucleic Acids Res. 2012;40(Database issue):D144–9. 10.1093/nar/gkr965 PMC3245155 22080564

[11] Lang D, Weiche B, Timmerhaus G, Richardt S, Riano-Pachon DM, Correa LGG, et al. Genome-Wide Phylogenetic Comparative Analysis of Plant Transcriptional Regulation: A Timeline of Loss, Gain, Expansion, and Correlation with Complexity. Genome Biol Evol. 2010;2:488–503. 10.1093/gbe/evq032 PMC2997552 20644220

[12] Pérez-Rodríguez P, Riaño-Pachón DM, Corrêa LGG, Rensing SA, Kersten B, Mueller-Roeber B (2010). PlnTFDB: updated content and new features of the plant transcription factor database. Nucleic Acids Res.

[13] Jin J, Zhang H, Kong L, Gao G, Luo J (2014). PlantTFDB 3.0: a portal for the functional and evolutionary study of plant transcription factors. Nucleic Acids Res.

[14] Charoensawan V, Wilson D, Teichmann SA (2010). Genomic repertoires of DNA-binding transcription factors across the tree of life. Nucleic Acids Res.

[15] Sharma N, Bhalla PL, Singh MB (2013). Transcriptome-wide profiling and expression analysis of transcription factor families in a liverwort, Marchantia polymorpha. BMC Genomics.

[16] Delwiche CF (1999). Tracing the Thread of Plastid Diversity through the Tapestry of Life. Am Nat.

[17] Keeling PJ (2004). Diversity and evolutionary history of plastids and their hosts. Am J Bot.

[18] Archibald JM (2009). The puzzle of plastid evolution. Curr Biol CB.

[19] Moustafa A, Beszteri B, Maier UG, Bowler C, Valentin K, Bhattacharya D (2009). Genomic Footprints of a Cryptic Plastid Endosymbiosis in Diatoms. Science.

[20] Wang D, Ning K, Li J, Hu J, Han D, Wang H, et al. Nannochloropsis Genomes Reveal Evolution of Microalgal Oleaginous Traits. PLoS Genet. 2014;10:e1004094. 10.1371/journal.pgen.1004094 PMC3886936 24415958

[21] Burki F, Okamoto N, Pombert J-F, Keeling PJ (2012). The evolutionary history of haptophytes and cryptophytes: phylogenomic evidence for separate origins. Proc Biol Sci.

[22] Andersen RA (2004). Biology and systematics of heterokont and haptophyte algae. Am J Bot.

[23] Iida K, Seki M, Sakurai T, Satou M, Akiyama K, Toyoda T, et al. RARTF: database and tools for complete sets of Arabidopsis transcription factors. DNA Res Int J Rapid Publ Rep Genes Genomes. 2005;12:247–56. 10.1093/dnares/dsi011 16769687

[24] Riaño-Pachón DM, Ruzicic S, Dreyer I, Mueller-Roeber B (2007). PlnTFDB: an integrative plant transcription factor database. BMC Bioinformatics.

[25] Guo A-Y, Chen X, Gao G, Zhang H, Zhu Q-H, Liu X-C, et al. PlantTFDB: a comprehensive plant transcription factor database. Nucleic Acids Res. 2008;36(Database issue):D966–9. 10.1093/nar/gkm841 PMC2238823 17933783

[26] Messina DN, Glasscock J, Gish W, Lovett M (2004). An ORFeome-based Analysis of Human Transcription Factor Genes and the Construction of a Microarray to Interrogate Their Expression. Genome Res.

[27] Adams MD, Celniker SE, Holt RA, Evans CA, Gocayne JD, Amanatides PG, et al. The Genome Sequence of Drosophila melanogaster. Science. 2000;287:2185–95. 10.1126/science.287.5461.2185 10731132

[28] Doebley J, Lukens L (1998). Transcriptional Regulators and the Evolution of Plant Form. Plant Cell Online.

[29] Lespinet O, Wolf YI, Koonin EV, Aravind L (2002). The role of lineage-specific gene family expansion in the evolution of eukaryotes. Genome Res.

[30] Nitta KR, Jolma A, Yin Y, Morgunova E, Kivioja T, Akhtar J, et al. Conservation of transcription factor binding specificities across 600 million years of bilateria evolution. eLife. 2015;4:e04837. 10.7554/eLife.04837 PMC4362205 25779349

[31] Carroll SB (2001). Chance and necessity: the evolution of morphological complexity and diversity. Nature.

[32] Van Nimwegen E (2003). Scaling laws in the functional content of genomes. Trends Genet.

[33] Vogel C, Chothia C (2006). Protein Family Expansions and Biological Complexity. PLoS Comput Biol.

[34] Wendel JF (2000). Genome evolution in polyploids. Plant Mol Biol.

[35] Paterson AH, Chapman BA, Kissinger JC, Bowers JE, Feltus FA, Estill JC (2006). Many gene and domain families have convergent fates following independent whole-genome duplication events in Arabidopsis, Oryza, Saccharomyces and Tetraodon. Trends Genet TIG.

[36] Edger PP, Pires JC (2009). Gene and genome duplications: the impact of dosage-sensitivity on the fate of nuclear genes. Chromosome Res Int J Mol Supramol Evol Asp Chromosome Biol.

[37] Carretero-Paulet L, Galstyan A, Roig-Villanova I, Martínez-García JF, Bilbao-Castro JR, Robertson DL (2010). Genome-Wide Classification and Evolutionary Analysis of the bHLH Family of Transcription Factors in Arabidopsis, Poplar, Rice, Moss, and Algae. Plant Physiol.

[38] Shalchian-Tabrizi K, Reier-Røberg K, Ree DK, Klaveness D, Bråte J (2011). Marine-freshwater colonizations of haptophytes inferred from phylogeny of environmental 18S rDNA sequences. J Eukaryot Microbiol.

[39] Bendif EM, Probert I, Schroeder DC, de Vargas C (2013). On the description of Tisochrysis lutea gen. nov. sp. nov. and Isochrysis nuda sp. nov. in the Isochrysidales, and the transfer of Dicrateria to the Prymnesiales (Haptophyta). J Appl Phycol.

[40] Schönknecht G, Chen W-H, Ternes CM, Barbier GG, Shrestha RP, Stanke M, et al. Gene transfer from bacteria and archaea facilitated evolution of an extremophilic eukaryote. Science. 2013;339:1207–10. 10.1126/science.1231707 23471408

[41] Inzé D, De Veylder L (2006). Cell Cycle Regulation in Plant Development 1. Annu Rev Genet.

[42] Ito M, Araki S, Matsunaga S, Itoh T, Nishihama R, Machida Y, et al. G2/M-phase-specific transcription during the plant cell cycle is mediated by c-Myb-like transcription factors. Plant Cell. 2001;13:1891–905. 10.1105/TPC.010102 PMC139135 11487700

[43] Yoshioka S, Taniguchi F, Miura K, Inoue T, Yamano T, Fukuzawa H (2004). The novel Myb transcription factor LCR1 regulates the CO2-responsive gene Cah1, encoding a periplasmic carbonic anhydrase in Chlamydomonas reinhardtii. Plant Cell.

[44] Zhao L, Gao L, Wang H, Chen X, Wang Y, Yang H, et al. The R2R3-MYB, bHLH, WD40, and related transcription factors in flavonoid biosynthesis. Funct Integr Genomics. 2013;13:75–98. 10.1007/s10142-012-0301-4 23184474

[45] Pattanaik S, Patra B, Singh SK, Yuan L (2014). An overview of the gene regulatory network controlling trichome development in the model plant, Arabidopsis. Front Plant Sci.

[46] Allison LA (2000). The role of sigma factors in plastid transcription. Biochimie.

[47] De J, Lai WS, Thorn JM, Goldsworthy SM, Liu X, Blackwell TK, Blackshear PJ (1999). Identification of four CCCH zinc finger proteins in Xenopus, including a novel vertebrate protein with four zinc fingers and severely restricted expression. Gene.

[48] Chai G, Hu R, Zhang D, Qi G, Zuo R, Cao Y, et al. Comprehensive analysis of CCCH zinc finger family in poplar (Populus trichocarpa). BMC Genomics. 2012;13:253. 10.1186/1471-2164-13-253 PMC3427045 22708723

[49] Yeh P-A, Yang W-H, Chiang P-Y, Wang S-C, Chang M-S, Chang C-J (2012). Drosophila eyes absent is a novel mRNA target of the tristetraprolin (TTP) protein DTIS11. Int J Biol Sci.

[50] Peng X, Zhao Y, Cao J, Zhang W, Jiang H, Li X, et al. CCCH-type zinc finger family in maize: genome-wide identification, classification and expression profiling under abscisic acid and drought treatments. PLoS ONE. 2012;7:e40120. 10.1371/journal.pone.0040120 PMC3391233 22792223

[51] Deng H, Liu H, Li X, Xiao J, Wang S (2012). A CCCH-type zinc finger nucleic acid-binding protein quantitatively confers resistance against rice bacterial blight disease. Plant Physiol.

[52] Schaffer R, Ramsay N, Samach A, Corden S, Putterill J, Carré IA, Coupland G (1998). The late elongated hypocotyl Mutation of Arabidopsis Disrupts Circadian Rhythms and the Photoperiodic Control of Flowering. Cell.

[53] Ehrenkaufer GM, Hackney JA, Singh U (2009). A developmentally regulated Myb domain protein regulates expression of a subset of stage-specific genes in Entamoeba histolytica. Cell Microbiol.

[54] Miyoshi K, Ito Y, Serizawa A, Kurata N (2003). OsHAP3 genes regulate chloroplast biogenesis in rice. Plant J.

[55] Combier J-P, Frugier F, de Billy F, Boualem A, El-Yahyaoui F, Moreau S, Vernié T, Ott T, Gamas P, Crespi M, Niebel A (2006). MtHAP2-1 is a key transcriptional regulator of symbiotic nodule development regulated by microRNA169 in Medicago truncatula. Genes Dev.

[56] Warpeha KM, Upadhyay S, Yeh J, Adamiak J, Hawkins SI, Lapik YR, Anderson MB, Kaufman LS (2007). The GCR1, GPA1, PRN1, NF-Y Signal Chain Mediates Both Blue Light and Abscisic Acid Responses in Arabidopsis. Plant Physiol.

[57] Cai X, Ballif J, Endo S, Davis E, Liang M, Chen D, DeWald D, Kreps J, Zhu T, Wu Y (2007). A Putative CCAAT-Binding Transcription Factor Is a Regulator of Flowering Timing in Arabidopsis. Plant Physiol.

[58] Nelson DE, Repetti PP, Adams TR, Creelman RA, Wu J, Warner DC, Anstrom DC, Bensen RJ, Castiglioni PP, Donnarummo MG, Hinchey BS, Kumimoto RW, Maszle DR, Canales RD, Krolikowski KA, Dotson SB, Gutterson N, Ratcliffe OJ, Heard JE (2007). Plant nuclear factor Y (NF-Y) B subunits confer drought tolerance and lead to improved corn yields on water-limited acres. Proc Natl Acad Sci.

[59] Mu J, Tan H, Zheng Q, Fu F, Liang Y, Zhang J, et al. LEAFY COTYLEDON1 Is a Key Regulator of Fatty Acid Biosynthesis in Arabidopsis. Plant Physiol. 2008;148:1042–54. 10.1104/pp.108.126342 PMC2556827 18689444

[60] Frontini M, Imbriano C, Manni I, Mantovani R (2004). Cell cycle regulation of NF-YC nuclear localization. Cell Cycle Georget Tex.

[61] Kahle J, Baake M, Doenecke D, Albig W (2005). Subunits of the Heterotrimeric Transcription Factor NF-Y Are Imported into the Nucleus by Distinct Pathways Involving Importin β and Importin 13. Mol Cell Biol.

[62] Wenkel S, Turck F, Singer K, Gissot L, Gourrierec JL, Samach A, Coupland G (2006). CONSTANS and the CCAAT Box Binding Complex Share a Functionally Important Domain and Interact to Regulate Flowering of Arabidopsis. Plant Cell Online.

[63] Yamamoto A, Kagaya Y, Toyoshima R, Kagaya M, Takeda S, Hattori T (2009). Arabidopsis NF-YB subunits LEC1 and LEC1-LIKE activate transcription by interacting with seed-specific ABRE-binding factors. Plant J.

[64] Jacquemin J, Ammiraju JSS, Haberer G, Billheimer DD, Yu Y, Liu LC, Rivera LF, Mayer K, Chen M, Wing RA (2014). Fifteen million years of evolution in the Oryza genus shows extensive gene family expansion. Mol Plant.

[65] Tanaka T, Maeda Y, Veluchamy A, Tanaka M, Abida H, Maréchal E, E, Bowler C, Muto M, Sunaga Y, Tanaka M, Yoshino T, Taniguchi T, Fukuda Y, Nemoto M, Matsumoto M, Wong PS, Aburatani S, Fujibuchi W: Oil accumulation by the oleaginous diatom Fistulifera solaris as revealed by the genome and transcriptome. Plant Cell. 2015;27:162–76. 10.1105/tpc.114.135194 PMC4330590 25634988

[66] Shiu S-H, Shih M-C, Li W-H (2005). Transcription Factor Families Have Much Higher Expansion Rates in Plants than in Animals. Plant Physiol.

[67] Kersting AR, Bornberg-Bauer E, Moore AD, Grath S (2012). Dynamics and adaptive benefits of protein domain emergence and arrangements during plant genome evolution. Genome Biol Evol.

[68] Khanna R, Kronmiller B, Maszle DR, Coupland G, Holm M, Mizuno T, Wu S-H (2009). The Arabidopsis B-box zinc finger family. Plant Cell.

[69] Kumagai T, Ito S, Nakamichi N, Niwa Y, Murakami M, Yamashino T, Mizuno T (2008). The common function of a novel subfamily of B-Box zinc finger proteins with reference to circadian-associated events in Arabidopsis thaliana. Biosci Biotechnol Biochem.

[70] Crocco CD, Holm M, Yanovsky MJ, Botto JF (2011). Function of B-BOX under shade. Plant Signal Behav.

[71] Huang J, Zhao X, Weng X, Wang L, Xie W (2012). The rice B-box zinc finger gene family: genomic identification, characterization, expression profiling and diurnal analysis. PLoS ONE.

[72] Bowler C, Botto J, Deng X-W (2013). Photomorphogenesis, B-Box Transcription Factors, and the Legacy of Magnus Holm. Plant Cell.

[73] Gregis V, Sessa A, Colombo L, Kater MM (2008). AGAMOUS-LIKE24 and SHORT VEGETATIVE PHASE determine floral meristem identity in Arabidopsis. Plant J.

[74] Immink RGH, Posé D, Ferrario S, Ott F, Kaufmann K, Valentim FL, Folter S de, Wal F van der, Dijk ADJ van, Schmid M, Angenent GC: Characterization of SOC1’s Central Role in Flowering by the Identification of Its Upstream and Downstream Regulators. Plant Physiol. 2012;160:433–49. 10.1104/pp.112.202614 PMC3440217 22791302

[75] Maejima K, Iwai R, Himeno M, Komatsu K, Kitazawa Y, Fujita N, Ishikawa K, Fukuoka M, Minato N, Yamaji Y, Oshima K, Namba S (2014). Recognition of floral homeotic MADS domain transcription factors by a phytoplasmal effector, phyllogen, induces phyllody. Plant J.

[76] Kewley RJ, Whitelaw ML, Chapman-Smith A (2004). The mammalian basic helix–loop–helix/PAS family of transcriptional regulators. Int J Biochem Cell Biol.

[77] Lindebro MC, Poellinger L, Whitelaw ML (1995). Protein-protein interaction via PAS domains: role of the PAS domain in positive and negative regulation of the bHLH/PAS dioxin receptor-Arnt transcription factor complex. EMBO J.

[78] Erbel PJA, Card PB, Karakuzu O, Bruick RK, Gardner KH (2003). Structural basis for PAS domain heterodimerization in the basic helix--loop--helix-PAS transcription factor hypoxia-inducible factor. Proc Natl Acad Sci U S A.

[79] Takahashi F, Yamagata D, Ishikawa M, Fukamatsu Y, Ogura Y, Kasahara M, Kiyosue T, Kikuyama M, Wada M, Kataoka H (2007). AUREOCHROME, a photoreceptor required for photomorphogenesis in stramenopiles. Proc Natl Acad Sci U S A.

[80] Ishikawa M, Takahashi F, Nozaki H, Nagasato C, Motomura T, Kataoka H (2009). Distribution and phylogeny of the blue light receptors aureochromes in eukaryotes. Planta.

[81] Vieler A, Wu G, Tsai C-H, Bullard B, Cornish AJ, Harvey C, Reca I-B, Thornburg C, Achawanantakun R, Buehl CJ, Campbell MS, Cavalier D, Childs KL, Clark TJ, Deshpande R, Erickson E, Armenia Ferguson A, Handee W, Kong Q, Li X, Liu B, Lundback S, Peng C, Roston RL, Sanjaya, Simpson JP, TerBush A, Warakanont J, Zäuner S, Farre EM (2012). Genome, Functional Gene Annotation, and Nuclear Transformation of the Heterokont Oleaginous Alga Nannochloropsis oceanica CCMP1779. PLoS Genet.

[82] Schellenberger Costa B, Sachse M, Jungandreas A, Bartulos CR, Gruber A, Jakob T, et al. Aureochrome 1a is Involved in the Photoacclimation of the Diatom Phaeodactylum tricornutum. PLoS ONE. 2013;8:e74451. 10.1371/journal.pone.0074451 PMC3779222 24073211

[83] Austin RW, Petzold TJ (1986). Spectral Dependence of the Diffuse Attenuation Coefficient of Light in Ocean Waters. Opt Eng.

[84] Huysman MJJ, Fortunato AE, Matthijs M, Costa BS, Vanderhaeghen R, Van den Daele H, Sachse M, Inzé D, Bowler C, Kroth PG, Wilhelm C, Falciatore A, Vyverman W, De Veylder L (2013). AUREOCHROME1a-mediated induction of the diatom-specific cyclin dsCYC2 controls the onset of cell division in diatoms (Phaeodactylum tricornutum). Plant Cell.

[85] Hegemann P (2008). Algal Sensory Photoreceptors. Annu Rev Plant Biol.

[86] Briggs WR, Christie JM (2002). Phototropins 1 and 2: versatile plant blue-light receptors. Trends Plant Sci.

[87] Christie JM, Blackwood L, Petersen J, Sullivan S (2015). Plant Flavoprotein Photoreceptors. Plant Cell Physiol.

[88] Feller A, Machemer K, Braun EL, Grotewold E (2011). Evolutionary and comparative analysis of MYB and bHLH plant transcription factors. Plant J Cell Mol Biol.

[89] Taylor BL, Zhulin IB (1999). PAS Domains: Internal Sensors of Oxygen, Redox Potential, and Light. Microbiol Mol Biol Rev.

[90] Miller G, Mittler R (2006). Could heat shock transcription factors function as hydrogen peroxide sensors in plants?. Ann Bot.

[91] Liu Y, Zhang C, Chen J, Guo L, Li X, Li W, Yu Z, Deng J, Zhang P, Zhang K, Zhang L (2013). Arabidopsis heat shock factor HsfA1a directly senses heat stress, pH changes, and hydrogen peroxide via the engagement of redox state. Plant Physiol Biochem PPB Société Fr Physiol Végétale.

[92] Partch CL, Gardner KH (2010). Coactivator recruitment: a new role for PAS domains in transcriptional regulation by the bHLH-PAS family. J Cell Physiol.

[93] Liu T, Golden JW, Giedroc DP (2005). A zinc(II)/lead(II)/cadmium(II)-inducible operon from the Cyanobacterium anabaena is regulated by AztR, an alpha3N ArsR/SmtB metalloregulator. Biochemistry (Mosc).

[94] Lavoie BD, Shaw GS, Millner A, Chaconas G (1996). Anatomy of a Flexer–DNA Complex inside a Higher-Order Transposition Intermediate. Cell.

[95] Aki T, Adhya (1997). Repressor induced site-specific binding of HU for transcriptional regulation. EMBO J.

[96] Santos JM, Freire P, Vicente M, Arraiano CM (1999). The stationary-phase morphogene bolA from Escherichia coli is induced by stress during early stages of growth. Mol Microbiol.

[97] Maris AE, Sawaya MR, Kaczor-Grzeskowiak M, Jarvis MR, Bearson SMD, Kopka ML, et al. Dimerization allows DNA target site recognition by the NarL response regulator. Nat Struct Mol Biol. 2002;9:771–8. 10.1038/nsb845 12352954

[98] Chai Y, Winans SC (2004). Site-directed mutagenesis of a LuxR-type quorum-sensing transcription factor: alteration of autoinducer specificity. Mol Microbiol.

[99] Cangiano G, Mazzone A, Baccigalupi L, Isticato R, Eichenberger P, De Felice M, et al. Direct and indirect control of late sporulation genes by GerR of Bacillus subtilis. J Bacteriol. 2010;192:3406–13. 10.1128/JB.00329-10 PMC2897654 20435725

[100] Takahashi Y, Yamaguchi O, Omata T (2004). Roles of CmpR, a LysR family transcriptional regulator, in acclimation of the cyanobacterium Synechococcus sp. strain PCC 7942 to low-CO(2) and high-light conditions. Mol Microbiol.

[101] Frías JE, Flores E, Herrero A (2000). Activation of the Anabaena nir operon promoter requires both NtcA (CAP family) and NtcB (LysR family) transcription factors. Mol Microbiol.

[102] Kawamukai M, Utsumi R, Takeda K, Higashi A, Matsuda H, Choi YL, et al. Nucleotide sequence and characterization of the sfs1 gene: sfs1 is involved in CRP*-dependent mal gene expression in Escherichia coli. J Bacteriol. 1991;173:2644–8. 10.1128/jb.173.8.2644-2648.1991 PMC207832 2013578

[103] Martin W, Rujan T, Richly E, Hansen A, Cornelsen S, Lins T (2002). Evolutionary analysis of Arabidopsis, cyanobacterial, and chloroplast genomes reveals plastid phylogeny and thousands of cyanobacterial genes in the nucleus. Proc. Natl. Acad. Sci. U.S.A..

[104] Leliaert F, Smith DR, Moreau H, Herron MD, Verbruggen H, Delwiche CF (2012). Phylogeny and Molecular Evolution of the Green Algae. Critical Reviews in Plant Sciences..

[105] Nosenko T, Bhattacharya D (2007). Horizontal gene transfer in chromalveolates. BMC Evol Biol.

[106] MacPherson S, Larochelle M, Turcotte B (2006). A Fungal Family of Transcriptional Regulators: the Zinc Cluster Proteins. Microbiol Mol Biol Rev.

[107] Todd RB, Andrianopoulos A (1997). Evolution of a fungal regulatory gene family: the Zn(II)2Cys6 binuclear cluster DNA binding motif. Fungal Genet Biol FG B.

[108] Pan T, Coleman JE (1990). GAL4 transcription factor is not a “zinc finger” but forms a Zn(II)2Cys6 binuclear cluster. Proc Natl Acad Sci U S A.

[109] Martens JA, Laprade L, Winston F (2004). Intergenic transcription is required to repress the Saccharomyces cerevisiae SER3 gene. Nature.

[110] Moye-Rowley WS (2003). Transcriptional control of multidrug resistance in the yeast Saccharomyces. Prog Nucleic Acid Res Mol Biol.

[111] Felenbok B, Flipphi M, Nikolaev I (2001). Ethanol catabolism in Aspergillus nidulans: a model system for studying gene regulation. Prog Nucleic Acid Res Mol Biol.

[112] Hynes MJ, Murray SL, Duncan A, Khew GS, Davis MA (2006). Regulatory genes controlling fatty acid catabolism and peroxisomal functions in the filamentous fungus Aspergillus nidulans. Eukaryot Cell.

[113] Garrido SM, Kitamoto N, Watanabe A, Shintani T, Gomi K (2012). Functional analysis of FarA transcription factor in the regulation of the genes encoding lipolytic enzymes and hydrophobic surface binding protein for the degradation of biodegradable plastics in Aspergillus oryzae. J Biosci Bioeng.

[114] McFadden GI (2014). Origin and Evolution of Plastids and Photosynthesis in Eukaryotes. Cold Spring Harb Perspect Biol.

[115] Richards TA, Soanes DM, Foster PG, Leonard G, Thornton CR, Talbot NJ (2009). Phylogenomic Analysis Demonstrates a Pattern of Rare and Ancient Horizontal Gene Transfer between Plants and Fungi. Plant Cell Online.

[116] Chan CX, Reyes-Prieto A, Bhattacharya D (2011). Red and green algal origin of diatom membrane transporters: insights into environmental adaptation and cell evolution. PLoS ONE.

[117] Mackiewicz P, Bodył A, Moszczyński K (2013). The case of horizontal gene transfer from bacteria to the peculiar dinoflagellate plastid genome. Mob Genet Elem.

[118] Qiu H, Yoon HS, Bhattacharya D (2013). Algal endosymbionts as vectors of horizontal gene transfer in photosynthetic eukaryotes. Plant Physiol.

[119] Qiu H, Price DC, Weber APM, Reeb V, Chan Yang E, Lee JM, et al. Adaptation through horizontal gene transfer in the cryptoendolithic red alga Galdieria phlegrea. Curr Biol. 2013;23:R865–6. 10.1016/j.cub.2013.08.046 24112977

[120] Beck A, Divakar PK, Zhang N, Molina MC, Struwe L (2014). Evidence of ancient horizontal gene transfer between fungi and the terrestrial alga Trebouxia. Org Divers Evol..

[121] Liu H, Probert I, Uitz J, Claustre H, Aris-Brosou S, Frada M, et al. Extreme diversity in noncalcifying haptophytes explains a major pigment paradox in open oceans. Proc Natl Acad Sci U S A. 2009;106:12803–8. 10.1073/pnas.0905841106 PMC2722306 19622724

[122] Krogh A, Brown M, Mian IS, Sjölander K, Haussler D (1994). Hidden Markov models in computational biology. Applications to protein modeling. J Mol Biol.

[123] Jones P, Binns D, Chang H-Y, Fraser M, Li W, McAnulla C, et al. InterProScan 5: genome-scale protein function classification. Bioinforma Oxf Engl. 2014;30:1236–40. 10.1093/bioinformatics/btu031 PMC3998142 24451626

[124] Finn RD, Clements J, Eddy SR (2011). HMMER web server: interactive sequence similarity searching. Nucleic Acids Res.

